# Sexual Function After Vaginal Delivery in Primiparous Women: A Perspective in the First Months Postpartum

**DOI:** 10.3390/healthcare13050566

**Published:** 2025-03-05

**Authors:** Silvio Tartaglia, Ludovica Puri, Francesca Brugnoli, Federico Quintiliani, Camilla Allegrini, Vitalba Gallitelli, Valentina Esposito, Marco De Santis, Daniela Visconti

**Affiliations:** 1 Ostetricia e Patologia Ostetrica, Dipartimento di Scienze della Salute della Donna, Fondazione Policlinico Universitario A. Gemelli IRCCS, del Bambino e di Sanità Pubblica, 00168 Rome, Italy; silvio.tartaglia@policlinicogemelli.it (S.T.); camilla.allegrini@policlinicogemelli.it (C.A.); valentina.esposito@policlinicogemelli.it (V.E.); marco.desantis@unicatt.it (M.D.S.); daniela.visconti@policlinicogemelli.it (D.V.); 2 Istituto di Clinica Ostetrica e Ginecologica, Università Cattolica del Sacro Cuore, 00168 Rome, Italy; ludovica.puri91@gmail.com (L.P.); federico23.quintiliani@gmail.com (F.Q.); 3 Dipartimento di Ginecologia e Ostetricia, Ospedale Isola Tiberina—Gemelli Isola, 00186 Rome, Italy; vitalbagallitelli@gmail.com

**Keywords:** female sexual dysfunction, sexual health, perineal tears, childbearing, FSFI

## Abstract

**Background/Objectives:** Female sexual dysfunction (FSD) involves persistent issues with desire, arousal, orgasm, or pain during intercourse. The Female Sexual Function Index (FSFI), a validated 19-item questionnaire, is widely used to assess FSD. Childbirth, particularly vaginal delivery with perineal trauma, can increase FSD risk, with 41–83% of women affected at six months postpartum. However, early postpartum FSD remains underexplored. This study examines FSD risk factors in first-time mothers delivering vaginally, using longitudinal FSFI assessments before and after the delivery. **Methods:** A prospective observational study was conducted involving 80 primiparous women who delivered vaginally. The FSFI questionnaire was provided before childbirth and three months postpartum. We compared the group of women who developed early FSD after delivery (N = 45) with those with a normal FSFI score (>26.6). **Results:** Three months after vaginal delivery, participants exhibited a significant decrease in overall FSFI scores (−9.61 [95%CI: −11.6; −7.6]; *p* = 0.008). A total of 45 patients (56.2%) developed early FSD. Marital status emerged as a significant factor, with marriage acting as a protective factor (OR 0.27 [95%CI 0.05–1.24]; *p* = 0.044). Clitoral and periclitoral tears were associated with a higher risk of FSD than high-degree perineal lacerations (OR 3.02 [95%CI 1.56–6.24]; *p* = 0.021). **Conclusions:** At three months post vaginal delivery, primiparous women face a relevant risk of developing transient sexual dysfunction. Marital status and type of perineal tears are identified as key factors influencing postpartum sexual function. Further research is warranted to explore these factors comprehensively and provide timely clinical and psychological support to couples navigating the challenges of early family life.

## 1. Introduction

Female sexual dysfunction (FSD) is a multifactorial disorder, comprising anatomical, psychological, physiological, and social–interpersonal components. One of the most complete and exhaustive definitions is a “persistent/recurring decrease in sexual desire or arousal, the difficulty/inability to achieve an orgasm, and/or the feeling of pain during sexual intercourse” [1]. Several patient-reported tools and inventories have been proposed to date to examine sexual behavior and satisfaction [2,3,4]. A questionnaire about women’s sexual health, the Female Sexual Function Index (FSFI), was presented more than twenty years ago and has been largely validated [5]. It consists of a 19-item self-reported outcome measure evaluating FSD, divided into six different domains (desire, arousal, lubrication, orgasm, satisfaction, and pain).

Childbirth is an event that impacts women’s lifestyles and changes priorities, and it can be one of the causes of FSD, both during pregnancy and the postpartum period. Studies report a variable incidence of FSD (41–83%) six months after delivery [6]. Most of the affected women resume their normal sexual activity one year after childbirth [7]. Several studies have investigated the correlation between the mode of delivery and the development of FSD during the postpartum period, but the findings are often inconsistent and contrasting [6,7,8,9,10]. Since vaginal delivery is associated with perineal trauma and pelvic floor damage, it was proposed that this mode of birth might cause postpartum FSD with a worse outcome than cesarean section (CS) since the latter does not involve perineal trauma.

As a consequence of vaginal birth, up to 90% of women develop perineal tears or episiotomy [11]. Lacerations are classified into four degrees based on the involvement of the skin, mucosa, muscles, anal sphincters, and rectal mucosa [12]. Most studies to date have evaluated female sexual well-being after six or twelve months. However, less information has been provided regarding the earlier phase of the postpartum period, which is filled with changes and challenges for couples.

To evaluate the possible risk factors and their relevance in FSD development in the early postpartum period, we designed a prospective study enrolling women in their first pregnancy who gave birth vaginally, and who were subjected to an FSFI questionnaire before and three months after the delivery.

## 2. Materials and Methods

### 2.1. Study Design

This prospective longitudinal observational study was conducted from January 2022 to December 2023 at the Obstetrics Department of Woman, Child, and Public Health of Fondazione Policlinico Universitario A. Gemelli IRCCS, Rome, Italy. This study was accepted by the Departmental Ethics Committee of the Università Cattolica del Sacro Cuore of Rome and reviewed by an Institutional Review Board (Prot. ID 15181/23). All the participants were recruited at the hospitalization for delivery and gave written consent for participation and the publication of personal data. The design, methodology, data analysis, and reporting of results were conducted following the STROBE checklist to ensure transparency, accuracy, and completeness in reporting.

Primiparas, Caucasian women aged between 18 and 45 with no indications for elective cesarean section at the beginning of the active stage of labor were included. Either spontaneous or induced labor was allowed, while multiple pregnancies and non-cephalic fetal presentations were exclusion criteria. An initial questionnaire was delivered to collect the patients’ socio-demographic and obstetrical characteristics and measure their FSFI before childbirth. The questionnaire’s total score can vary from a minimum of 2 to a maximum of 36 and was calculated using conversion coefficients, as described in Table 1.

In our study, the first questionnaire consisted of two parts and was organized into multiple-choice answers. The first part included eight questions on socio-demographic data and obstetrics and neonatal details; the second part included 19 questions on sexual function preceding pregnancy and conception. Patients were invited to self-report their sexual function, referring to the period immediately before pregnancy. All the data inherent in the delivery and the newborn were collected for all the participants. In particular, the following characteristics were reported: the duration of the active stage and pushing, the mode of induction and rupture of membranes, the newborn’s dimensions, and the degree of perineal laceration, according to the literature [11]. Three months after delivery, a second web questionnaire was given anonymously to the participant who delivered vaginally and we confirmed their consent to participate. This second questionnaire consisted of 20 questions aimed to assess the resumption of sexual activity, their breastfeeding habit, and to calculate the FSFI postpartum. Female sexual dysfunction (FSD) was evaluated according to an FSFI threshold [5]. Values on the FSFI of 26.6 or lower indicated the presence of FSD, while values above 26.6 were considered descriptive of normal sexual function. Study data were anonymized and prospectively collected by the researchers officially involved.

### 2.2. Statistical Analysis

Results are presented as *n* (%) for categorical variables and mean ± Standard Deviation (SD) or median (min-max) for continuous variables as appropriate. The association between the socio-demographic, obstetric, newborn, and sexual dysfunction characteristics of the patients was assessed with Pearson’s Chi-Square test, dividing patients into two groups: those who presented with FSD three months after delivery and those who did not. The Chi-Square test was also used to calculate the odds ratio (OR) using 2 × 2 contingency tables for binary variables. Differences in baseline, obstetrics, and newborn characteristics were evaluated using Student’s *t*-test for continuous variables and McNemar’s test for categorical variables. Pre- and postpartum FSFI questionnaire items (desire, arousal, lubrication, orgasm, satisfaction, and pain) and FSFI scores were compared using paired Student’s *t*-test. Logistic regression analysis was also applied to evaluate the possible effect of some confounders on the FSD incidence. We included the potential confounding variables that resulted in a significant difference between the two groups. A *p*-value lower than 0.05 was considered statistically significant. No imputation was carried out for missing data. Statistical analysis was performed using SPSS Version 20 (Statistical Package for Social Science, Chicago, IL, USA).

## 3. Results

A total of 120 nulliparous women in the third trimester of pregnancy participated by completing the initial FSFI questionnaire. During labor, N = 11 women required an urgent cesarean section, while N = 10 underwent operative vaginal delivery, leading to the exclusion of 21 women from the next phase of the study. Additionally, N = 19 participants did not complete the postpartum assessment of sexual function following delivery. Every participant requested and received epidural analgesia during labor. Ultimately, N = 80 participants met the inclusion criteria and successfully completed the study by submitting a second questionnaire three months post delivery (Table 2).

The results of the FSFI questionnaires compiled before delivery (T0) and three months after delivery (T1) are shown in Table 3, divided by domain.

**Figure 1:**
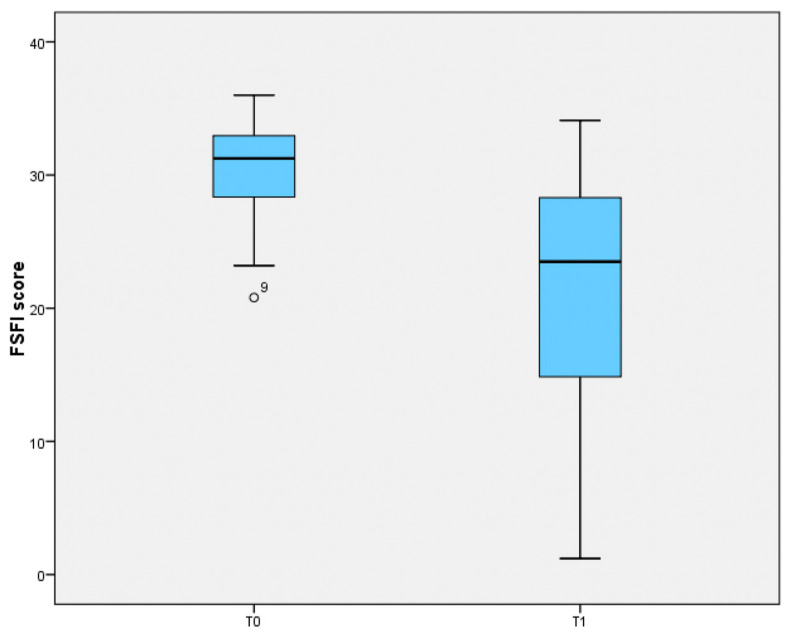
Comparison of FSFI score before (T0) and after delivery (T1).

Paired *t*-test on the overall FSFI results reported a statistically significant difference between the T0 and T1 evaluations (−9.61 [95%CI: −11.6; −7.6]; *p* = 0.008) (Figure 1).

**Figure 2:**
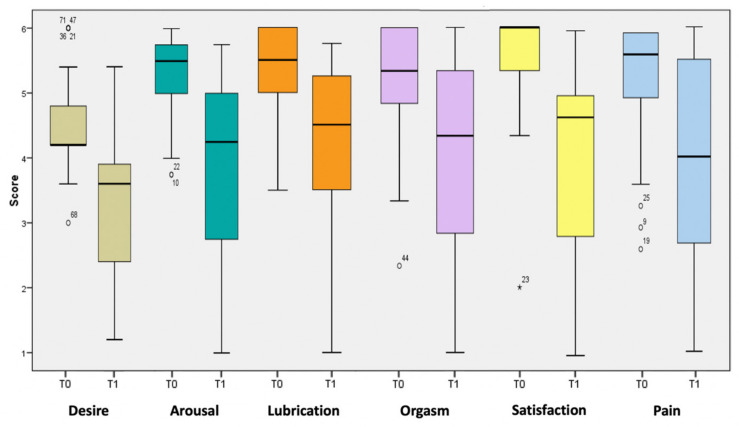
Boxplot of the score per FSFI item before (T0) and after delivery (T1).

Analyzing the single items composing the multifactorial questionnaire, the most negatively affected domains of the FSFI were “desire” (mean difference −1.29; *p* = 0.018), “orgasm” (−1.58; *p* = 0.001), and “pain” (−1.63; *p* = 0.043) (Figure 2).

After, the participants were divided into two groups according to their FSFI score at T1: the women who developed FSD (defined as an FSFI score <26.6) (*n* = 45) and women who had an adequate sexual score three months after delivery (*n* = 35). In the FSD group, 36 women (80%) resumed sexual activity within three months after delivery compared to 33 (94.3%) in the other group (*p* = 0.05).

Comparisons of the baseline characteristics, obstetrics, and neonatal data between the two groups are presented in Table 4.

No differences were retrieved regarding maternal age, BMI, weight gain, induction method, newborn measures, duration of labor, gestational age, profession, and education. The only statistically significant differences between the two groups arose from the comparison of marital status and the type of vulvar–perineal lacerations.

Multivariate logistic regression confirmed the results of the initial comparison between the group of women who developed an FSD three months after delivery and women who did not. Being married resulted in the most relevant protective factor for FSD after birth (OR 0.27 [95%CI 0.05–1.24]; *p* = 0.044), while being unmarried seems to be a significant risk factor for developing sexual dysfunction in the first months after vaginal delivery (OR 2.96 [95%CI 1.69–5.31]; *p* = 0.012). First-degree lacerations involving the labia or clitoral region are statistically significantly correlated to the risk of FSD after delivery (OR 3.02 [95%CI 1.56–6.24]; *p* = 0.021), more so than those second-degree lacerations (OR 0.55 [95%CI 0.01–1.99]; *p* = 0.206).

## 4. Discussion

The postpartum period is marked by hormonal changes, sleep loss, fatigue, and stress stemming from the adjustment to the new role of the couple [9]. Additional factors contributing to postpartum FSD are a lack of interest in sex, body image changes, breastfeeding, postpartum depression, and a lack of time for intimacy [13,14]. Breastfeeding is characterized by several hormonal modifications that can relate to changes in sexuality. After a vaginal delivery, breastfeeding mothers experience elevated levels of prolactin, a hormone essential for milk production. This increase in prolactin suppresses the secretion of estrogen and androgens through negative feedback mechanisms, leading to hypoestrogenism. The resulting low estrogen levels can cause vaginal dryness and atrophy, contributing to discomfort or pain during sexual intercourse (dyspareunia) [15]. According to Adler et al., women reporting severe reduction in sexual interest are more frequently exposed to a reduction in testosterone and androstenedione levels, but there is no strong correlation to levels of prolactin or estrogen [16].

Many women experience perineal pain, dyspareunia, insufficient lubrication, painful orgasms or an inability to achieve them, lack of desire, post-coital bleeding, burning sensations, and reduced sexual satisfaction. Additionally, perineal tears from vaginal delivery can lead to pain during intercourse. It has been suggested that the extent of perineal injury during delivery significantly influences the timing and quality of the resumption of sexual activity in the postpartum period [17]. A recent meta-analysis found that the mode of delivery does not significantly affect postpartum sexual function [9]. Therefore, current evidence does not support the use of cesarean sections to protect postpartum sexual function [8,10]. The main distinction between vaginal delivery and cesarean section appears to be the risk of varying degrees of perineal trauma associated with the vaginal route. In a prospective multicenter cohort study conducted in Denmark, women who delivered with intact perineum or first-degree tears reported the best outcomes, while those with second-, third-, and fourth-degree tears (known as obstetric anal sphincter injuries, OASISs) faced more sexual difficulties in the years following childbirth and experienced a delayed return to sexual intercourse [18]. Both psychological, social, and clinical factors seem to play roles in the development of FSD after vaginal delivery [19].

In the present study, a group of homogeneous nulliparous women were asked to give feedback about their sexual behavior before giving birth and three months after delivery. The comparison of the FSFI questionnaire results depicts an overall worsening of sexual function (*p* = 0.008) and a high percentage of early FSD (56.2%) three months after birth. “Desire”, “orgasm”, and “pain” were the domains most negatively affected by vaginal delivery. Consistent with other findings, a significantly lower percentage of women presenting FSD resumed satisfying sexual activity within three months after delivery compared to those with adequate sexual status (*p* = 0.05). For a higher-than-expected percentage of women in our study population, the postpartum period is marked by a lack of sexual interest and painful intercourse with a prejudiced capacity to reach a satisfying orgasm. Other authors reported a less pronounced reduction in FSFI score one year after delivery. In the study by Zgliczynska et al., almost half of the women experienced a loss of at least 10% in FSFI score after delivery, while in our population the decrease is more evident (31%) [13]. Saydam and colleagues identified a higher incidence of FSD (67.7%) compared to our study (56.2%), but confirmed a statistically significant difference in FSFI score six months after delivery [20].

While psychosocial elements are likely involved in the first aspect, a physical component linked to vulvar and perineal tears could play a role in the latter. Analyzing the results of multivariate regression analysis, a statistically significant correlation in marital status resulted from the comparison between FSD incidence and the confounding factors analyzed. Being married seems to be a protective factor for the risk of FSD. At the same time, women who are unmarried or non-cohabiting with their partners significantly experienced early sexual dysfunction. Several studies have proved the relationship between FSD and marital satisfaction [21,22]. It has been speculated that married couples are more sexually satisfied than singles [23,24]. Marital distress has been indicated as a relevant risk factor for sexual dysfunction in both partners [25]. The results of our analysis seem to describe a postpartum-life couple condition that is positively affected by a stable, continuative, and committed couple relationship. The reason for this correlation could lie in the intimacy of a consolidated relationship or the domestic support received to cope with the maternity burdens that characterize the first months after childbirth.

The genesis of the orgasm is a complex, multifactorial process involving anatomical, neurological, hormonal, psychological, and personal components [26]. Anatomically, the female sexual response is marked by several physiological changes in the vulva, labia, and clitoris [27]. The clitoris serves as the core for the orgasmic response, with its sensitivity defined by a complex vascular and neuronal network that converges in the glans from the bulbs, roots, and crus [28]. Orgasm results in a significant increase in blood supply to the clitoris, causing volumetric enlargement. The resistance within the blood vessels of the clitoral and uterine arteries has been found to have a significant association with various cardiometabolic risk factors [29], as well as concerns related to body image [30], among women who seek medical consultation for sexual health symptoms. This relationship suggests that vascular health may play a crucial role in cardiovascular and metabolic conditions while potentially influencing psychological and emotional well-being in sexual function. In cases of periclitoral or periurethral tears that affect the delicate anatomy of the clitoris, the mechanism behind the increase in vascular supply to the clitoris may become dysfunctional, leading to pain during arousal.

Contrary to previous reports, in our population, lacerations higher than first degree are associated with a lower incidence of early female sexual dysfunction (FSD) compared to superficial spontaneous lacerations in the clitoral area [18]. A large retrospective analysis involving over 14,000 vaginal singleton deliveries indicated that epidural anesthesia is the only protective factor that reduces the risk of clitoral lacerations [31]. In our study, only women delivering under epidural analgesia were considered for the final analysis; however, our incidence (40%) was higher than the 0.5% reported in the study by Simek et al. This discrepancy likely arises from our decision to include all lacerations affecting the clitoris, labia, periclitoral, and periurethral regions in the same category. Pregnancy itself may contribute to a decrease in female sexual desire since the etiology of FSD is multifactorial [13]. This is particularly evident in the postpartum period. The initial months of becoming a first-time mother are filled with new and often overwhelming tasks related to baby care and recovery from delivery. Hormonal changes, breastfeeding, and maternity blues are just a few of the various factors that can lead to a decline in sexual interest and the ability to achieve orgasm. Fortunately, normal vaginal birth is typically associated with only a transient decrease in sensitivity, which often recovers spontaneously by twelve months [32].

Our study did not evaluate the male partner’s point of view, which could represent a limitation. No information has been collected about the newborn status, sleeping habits, or colic symptoms that might cause exhaustion in mothers and might be related to reduced sexual performance. Furthermore, the lack of a control group and the small population conditioned our results but represent a cue for future developments. The single postpartum evaluation of the population at three months after a vaginal delivery represents, at the same time, a weakness and a strength of our investigation. In carrying this out, we have no data about the eventual resumption of adequate sexual behavior after a longer period, although evaluation at six or twelve months has already been studied [6]. There are no consistent reports of the evaluation of the female sexual function at such a brief interval from the delivery. A very recent descriptive cross-sectional study investigated the resumption of sexual activity after birth in the first weeks postpartum [33]. Some of the participants filled out the provided questionnaire (ten questions about desire, arousal, lubrication, orgasm, satisfaction, pain, and knowledge and attitude about contraception) in the first 6 weeks after the delivery, but only 7.93% had resumed sexual activity during that period. The FSFI score was like our results, but in our population, 86.25% of participants resumed sexual intercourse, giving more reliability to our data. Another issue that should be addressed relates to the maternal body evaluation. We compared BMI and weight gain during pregnancy between the women who developed FSD after delivery and those who did not, with no statistically significant differences. Many studies confirmed the impact of visceral adiposity and perceptions of physical appearance on female sexual function, with a higher reliability grade than BMI alone [34,35]. While BMI has been widely used as a measure of adiposity, it does not fully capture visceral fat distribution, which has been shown to play a role in FSD. Although our study did not consider the assessment of visceral fat and anxiety related to physical appearance, it is desirable that future studies investigating FSD include and report these findings.

The relevance of our report lies just in the population enrolled and the timing of the FSFI questionnaire submission to provide additional evidence to the existing literature at a brief distance from delivery: this is an event that, at the same time, is a wonderful happening in the life of a “newborn family” and a potentially detrimental event for future female sexual health. Support for the affected women and their partners is aimed at education, cognitive behavior therapy, couple therapy, physiotherapy, and treatment of contributing factors [36]. Educating couples about possible transitory effects on anatomy and arousal after childbirth, recommending psychosexual counseling, and increasing insight into this very-often-silenced issue is vital for the couple to overcome the problem in the best possible way [37].

## 5. Conclusions

In conclusion, three months after a vaginal delivery, women face a significant risk of experiencing transient sexual dysfunction, which can impact their mental health and relationship with their partner. Being married, and indicating a stable and committed relationship, appears to be a protective factor. Furthermore, clitoral and periclitoral tears, although superficial and repaired, negatively influence women’s sexual life more than high-degree perineal lacerations or the size of the baby. Although the duration of labor relates to poorer FSFI scores following delivery, this relationship lacks statistical significance.

Further and larger epidemiological reports are needed to ascertain the weight of the single factors composing the birth event, to prevent, where feasible, potentially detrimental effects deriving from such joyful moments, and to provide couples with the due clinical and psychological support in the earliest months of their family life.

## Figures and Tables

**Table 1:** Criteria for Female Sexual Function Index calculation.

Items	Question	Score Range	Factor	Minimum Score	Maximum Score
Desire	1, 2	1–5	0.6	1.2	6
Arousal	3, 4, 5, 6	0–5	0.3	0	6
Lubrication	7, 8, 9, 10	0–5	0.3	0	6
Orgasm	11, 12, 13	0–5	0–4	0	6
Satisfaction	14, 15, 16	0 (or 1)–5	0.4	0.8	6
Pain	17, 18, 19	0–5	0–4	0	6
Full Scale	-	-	-	2	36

**Table 2:** Baseline characteristics of the population.

Variable	Population N = 80
Age (years)	35.7 ± 4.2
BMI at Admission (kg/m^2^)	24.5 ± 4.3
BMI at Delivery (kg/m^2^)	27.7 ± 4.9
Weight Gain (kg)	10.0 ± 4.0
**Marital Status (*n*, %)**	
Married	43 (53.7)
**Education (*n*, %)**	
Secondary School	5 (6.2)
High School	22 (27.5)
Graduation	53 (66.2)
**Profession (*n*, %)**	
Freelancer	11 (13.7)
Office Worker	49 (61.2)
Manager	3 (3.7)
Service and Sales Worker	4 (5.0)
Student	3 (3.7)
Unemployed	10 (12.5)

BMI, body mass index.

**Table 3:** Postpartum modification of the score per domain.

Items	Before Delivery (T0)	After Delivery (T1)	Mean Difference	*p*-Value
Desire	4.47 (±0.62)	3.18 (±1.1)	−1.29	0.018 *
Arousal	5.24 (±0.66)	3.41 (±1.8)	−1.84	0.135
Lubrication	5.2 (±0.72)	3.73 (±1.83)	−1.47	0.162
Orgasm	5.11 (±0.91)	3.53 (±2.03)	−1.58	0.001 *
Satisfaction	5.53 (±0.95)	3.68 (±1.94)	−1.85	0.467
Pain	5.17 (±0.95)	3.54 (±1.96)	−1.63	0.043 *
FSFI	30.67 (±3.1)	21.06 (±9.43)	−9.61	0.008 *

FSFI, Female Sexual Function Index. Results are presented as mean (±SD). *p*-values were calculated using paired Student’s *t*-test. * Statistically significant difference *p* < 0.05.

**Table 4:** Comparison of socio-demographic, obstetric, and neonatal variables between the two groups.

Variable	Early Postpartum FSD N = 45	No Postpartum FSD N = 35	*p*-Value
Age (years)	35.1 ± 4.1	36.3 ± 4.3	0.205
BMI at Admission (kg/m^2^)	24.1 ± 3.1	25.0 ± 5.6	0.145
BMI at Delivery (kg/m^2^)	27.5 ± 4.7	27.9 ± 5.1	0.234
Weight Gain (kg)	10.1 ± 3.9	9.9 ± 4.1	0.211
**Marital Status (*n*, %)**			
Married	18 (40)	25 (71)	0.01 *
**Education (*n*, %)**			
Secondary School	2 (4.4)	3 (8.6)	0.449
High School	13 (29.9)	9 (25.7)	0.752
Graduation	30 (66.7)	23 (65.7)	0.928
**Profession (*n*, %)**			
Freelancer	4 (8.9)	7 (20)	0.152
Office Worker	28 (62.2)	21 (60)	0.839
Manager	3 (6.7)	0 (0)	0.119
Service and Sales Worker	2 (4.4)	2 (5.7)	0.796
Student	2 (4.4)	1 (2.8)	0.710
Unemployed	6 (13.3)	4 (11.4)	0.798
GA at delivery (*n*, %)	39.5 ± 3.2	39.6 ± 2.9	0.819
**IOL (*n*, %)**			
Cervical Ripening	4 (8.9)	21 (60)	0.448
Prostaglandins	15 (33.3)	5 (14.3)	0.538
Oxytocin	3 (6.7)	14 (40)	0.861
AROM (*n*, %)	20 (44.4)	2 (5.7)	0.510
**Newborn**			
Weight (gr)	3265.3 ± 415.5	3295.6 ± 308.5	0.712
Head Circumference (mm)	34.4 ± 0.9	34.6 ± 0.8	0.250
**Labor Characteristics**			
Second Stage of Labor (min)	73.9 ± 46.5	62.2 ± 45.2	0.259
Active Pushing (min)	66.5 ± 33.2	57.8 ± 32.9	0.256
**Type of Laceration (*n*, %)**			
Intact Perineum	5 (11.1)	5 (14.3)	0.670
Labial/Periclitoral/Periurethral Tears	23 (51.1)	9 (25.7)	0.021 *
Grade II	12 (26.7)	14 (40)	0.206
Grade III/IV	0 (0)	2 (5.7)	0.104
Episiotomy	5 (11.1)	5 (14.3)	0.670
Breastfeeding (*n*, %)	27 (60)	22 (62.8)	0.794
**FSFI Score**			
Antepartum (T0)	30.8 ± 2.3	30.4 ± 3.9	0.618
Postpartum (T1)	16.6 ± 8.1	26.7 ± 7.8	<0.001 *
Resumption of Sexual Activity	36 (80)	33 (94.3)	0.05

BMI, body mass index; GA, gestational age; IOL, induction of labor; AROM, artificial rupture of membranes. Results are presented as *n* (%) or mean (± SD). *p*-values were calculated using Pearson’s Chi-Square two-sided test or Student’s *t*-test. * Statistically significant difference *p* < 0.05.

## Data Availability

The raw data supporting the conclusions of this article will be made available by the authors upon request.

## Abbreviations

The following abbreviations are used in this manuscript:

FSD	Female Sexual Disfunction
FSFI	Female Sexual Function Index
CS	Cesarean Section
BMI	Body Mass Index
GA	Gestational Age
IOL	Induction of Labor
AROM	Artificial Rupture of Membranes
